# Cardiovascular phenotype of the *Dmd^mdx^* rat – a suitable animal model for Duchenne muscular dystrophy

**DOI:** 10.1242/dmm.047704

**Published:** 2021-02-22

**Authors:** Petra Lujza Szabó, Janine Ebner, Xaver Koenig, Ouafa Hamza, Simon Watzinger, Sandra Trojanek, Dietmar Abraham, Hannes Todt, Helmut Kubista, Klaus Schicker, Séverine Remy, Ignacio Anegon, Attila Kiss, Bruno K. Podesser, Karlheinz Hilber

**Affiliations:** 1 Ludwig Boltzmann Institute for Cardiovascular Research at Center for Biomedical Research, Medical University of Vienna, Vienna 1090, Austria; 2 Department of Neurophysiology and Pharmacology, Center for Physiology and Pharmacology, Medical University of Vienna, Vienna 1090, Austria; 3 Center for Anatomy and Cell Biology, Medical University of Vienna, Vienna 1090, Austria; 4 INSERM, Center for Research in Transplantation and Immunology, UMR 1064, Nantes Université, F-44000 Nantes, France

**Keywords:** Muscular dystrophy, Remodeling, Cardiovascular dysfunction, Cardiomyocyte, Rat

## Abstract

Besides skeletal muscle abnormalities, Duchenne muscular dystrophy (DMD) patients present with dilated cardiomyopathy development, which considerably contributes to morbidity and mortality. Because the mechanisms responsible for the cardiac complications in the context of DMD are largely unknown, evidence-based therapy approaches are still lacking. This has increased the need for basic research efforts into animal models for DMD. Here, we characterized in detail the cardiovascular abnormalities of *Dmd^mdx^* rats, with the aim of determining the suitability of this recently established dystrophin-deficient small animal as a model for DMD.

Various methods were applied to compare cardiovascular properties between wild-type and *Dmd^mdx^* rats, and to characterize the *Dmd^mdx^* cardiomyopathy. These methods comprised echocardiography, invasive assessment of left ventricular hemodynamics, examination of adverse remodeling and endothelial cell inflammation, and evaluation of vascular function, employing wire myography. Finally, intracellular Ca^2+^ transient measurements, and recordings of currents through L-type Ca^2+^ channels were performed in isolated single ventricular cardiomyocytes. We found that, similar to respective observations in DMD patients, the hearts of *Dmd^mdx^* rats show significantly impaired cardiac function, fibrosis and inflammation, consistent with the development of a dilated cardiomyopathy. Moreover, in *Dmd^mdx^* rats, vascular endothelial function is impaired, which may relate to inflammation and oxidative stress, and Ca^2+^ handling in *Dmd^mdx^* cardiomyocytes is abnormal.

These findings indicate that *Dmd^mdx^* rats represent a promising small-animal model to elucidate mechanisms of cardiomyopathy development in the dystrophic heart, and to test mechanism-based therapies aiming to combat cardiovascular complications in DMD.

## Abstract (precis)

**Summary:** We characterized the cardiovascular abnormalities of *Dmd^mdx^* rats, demonstrating that *Dmd^mdx^* rats show similar cardiac and vascular endothelial function impairments to Duchenne muscular dystrophy patients, representing a model of the dystrophic heart.

## INTRODUCTION

Duchenne muscular dystrophy (DMD), induced by mutations in the gene encoding for the intracellular protein dystrophin, is a severe X chromosome-linked illness characterized by progressive muscle weakness and degeneration. Besides the well-characterized skeletal muscle pathology, DMD is also associated with relevant cardiac complications (Shirokova and Niggli, 2013; Spurney, 2011). Among those, cardiac arrhythmias and the development of a dilated cardiomyopathy considerably contribute to the morbidity and mortality concomitant with the disease. The mechanisms responsible for the cardiac complications in the context of DMD are largely unknown, and this has increased the need for basic research efforts into animal models for DMD.

Among the used DMD animal models (McGreevy et al., 2015; Wells, 2018), the mdx mouse is the best known and the most widely used. It has a premature stop mutation in exon 23 of the murine *Dmd* gene, and consequently fails to translate functional full-length dystrophin (Sicinski et al., 1989). Although the mdx mouse is a useful genetic and biochemical model of DMD, it only partially mimics the human disease. Thus, in contrast to DMD patients, mdx mice only have a slightly shortened life span and do not show obvious clinical signs of muscular dystrophy (Grady et al., 1997; Gutpell et al., 2015). Further, cardiac abnormalities in mdx mice only develop late (Quinlan et al., 2004), and the cardiomyopathy is mild compared to that occurring in DMD patients (Grady et al., 1997; Janssen et al., 2005). This questions the suitability of this animal model for studying the cardiac disease phenotype.

In 2014, Larcher and colleagues described the development of dystrophin-deficient rats using transcription activator-like effector nucleases targeting exon 23 of the *Dmd* gene (Larcher et al., 2014). In these *Dmd^mdx^* rats, cardiac muscle was affected by necrosis and fibrosis, and showed signs of progressive dilated cardiomyopathy. Echocardiography revealed significant concentric remodeling, and alteration of diastolic function. Based on these findings, the authors argued that the cardiac disease phenotype in *Dmd^mdx^* rats closely mimics that observed in DMD patients, and that this animal model is potentially suitable for preclinical DMD research (Larcher et al., 2014). A weakness of the study – with an actual focus on skeletal muscle – is that the cardiac disease phenotype of *Dmd^mdx^* rats was not characterized in great detail. For example, echocardiography was only performed on 3-month-old, but not older, rats. Moreover, the authors (Larcher et al., 2014) did not investigate potentially occurring vascular complications such as enhanced arterial stiffness (Ryan et al., 2017) and endothelial cell (EC) dysfunction (Miike et al., 1987), which may also contribute to the development of the cardiac disease phenotype in DMD patients. Finally, functional studies at the cellular level (i.e. on *Dmd^mdx^* cardiomyocytes) have not yet been performed. Considering this lack of evidence, the aim of the present study was to provide a detailed characterization of the cardiac and vascular abnormalities in *Dmd^mdx^* rats both at the organ and cellular level. Our results suggest that *Dmd^mdx^* rats closely mimic the cardiovascular phenotype of DMD patients, and can thus be considered a promising small-animal model for the human disease.

## RESULTS

### Animal characteristics

Table 1 provides the summary of the animal characteristics. There was a significant difference in body weight between *Dmd^mdx^* (*n*=14) and wild-type (wt) (*n*=15) rats at the age of 9 months (Table 1). In addition, the total heart weight was decreased in *Dmd^mdx^* (*n*=10) versus wt (*n*=11) rats. Moreover, the wet lung weight, as well as the lung weight to body weight ratio, were markedly increased in the *Dmd^mdx^* (*n*=10) versus wt (*n*=11) rats at the age of 9 months, indicating cardiac dysfunction (Table 1).

**Table 1.**
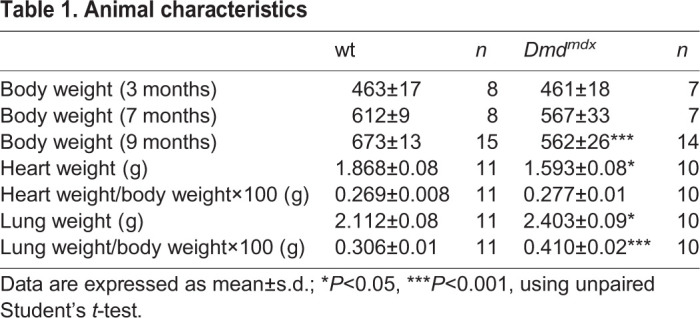
**Animal characteristics**

### Cardiac function and morphology

**Fig. 1.**
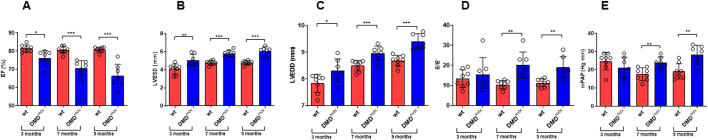
**Cardiac function and morphology assessment by transthoracic echocardiography.** (A-E) Ejection fraction (EF; A), left ventricular (LV) end-systolic diameter (LVESD; B), LV end-diastolic diameter (LVEDD; C), ratio between early mitral inflow velocity and mitral annular early diastolic velocity (E/E′; D) and mean pulmonary artery pressure (mPAP; E) in wt and *Dmd^mdx^* rats at 3, 7 and 9 months of age. Data are expressed as mean±s.d.; *n*=8 wt and *n*=7 *Dmd^mdx^*. *P<0.05, **P<0.01 and ***P<0.001, using unpaired Student's *t*-test at defined age (3, 7 and 9 months).

Left ventricular (LV) systolic and diastolic function, LV dimension and mean pulmonary artery pressure were assessed by transthoracic echocardiography (Fig. 1 and Table 2). We observed a slight, but significant, reduction in ejection fraction (EF) in *Dmd^mdx^* rats compared to wt rats at the age of 3 months (Fig. 1A). Moreover, EF progressively declined in *Dmd^mdx^* rats compared to wt rats at the age of 7 and 9 months (Fig. 1A). Conversely, *Dmd^mdx^* rats showed increased LV end-systolic (LVESD) and end-diastolic (LVEDD) diameters compared to wt rats, indicating progressive dilatation (Fig. 1B,C, respectively). At the age of 3 months, the *Dmd^mdx^* rats showed a tendency towards an increased ratio between early mitral inflow velocity and mitral annular early diastolic velocity **(**E/E′ ratio), becoming significant at 7 and 9 months (Fig. 1D), reflecting elevated LV filling pressure and LV diastolic dysfunction. Furthermore, mean pulmonary artery pressure (mPAP) was significantly increased in the *Dmd^mdx^* rats compared to wt rats at the age of 7 and 9 months (Fig. 1E), indicating pulmonary hypertension.

**Table 2.**
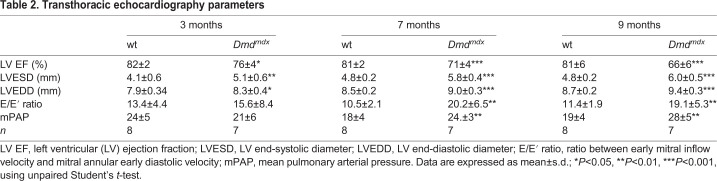
**Transthoracic echocardiography parameters**

### LV hemodynamic function

To complement the echocardiography data, we measured LV hemodynamic parameters by an invasive method in *Dmd^mdx^* (*n*=10) and wt (*n*=8) rats at the age of 9 months. *Dmd^mdx^* rats exhibited reduced LV systolic function, as demonstrated by a significant decrease in the maximal LV systolic pressure (74±3 mmHg versus 95±4 mmHg in wt; *P*=0.001) and also significantly reduced rate of contraction [maximal rate of LV pressure development (dP/dt_max_), 4458±344 mmHg/s versus 5956±288 mmHg/s in wt; *P*=0.004]. Simultaneously, LV end-diastolic pressure (LVEDP) was significantly increased in *Dmd^mdx^* rats compared to wt rats (4.8±0.5 mmHg versus 2.6±0.3 mmHg; *P*=0.0009). Similarly, the minimal rate of LV pressure fall (dP/dt_min_) was significantly lower and revealed diastolic dysfunction in *Dmd^mdx^* rats (−3071±276 mmHg/s versus −4464±339 mmHg/s in wt; *P*=0.006).

### Altered regulation of Ca^2+^ homeostasis, and oxidative and mechanical stress in dystrophic myocardium

**Fig. 2.**
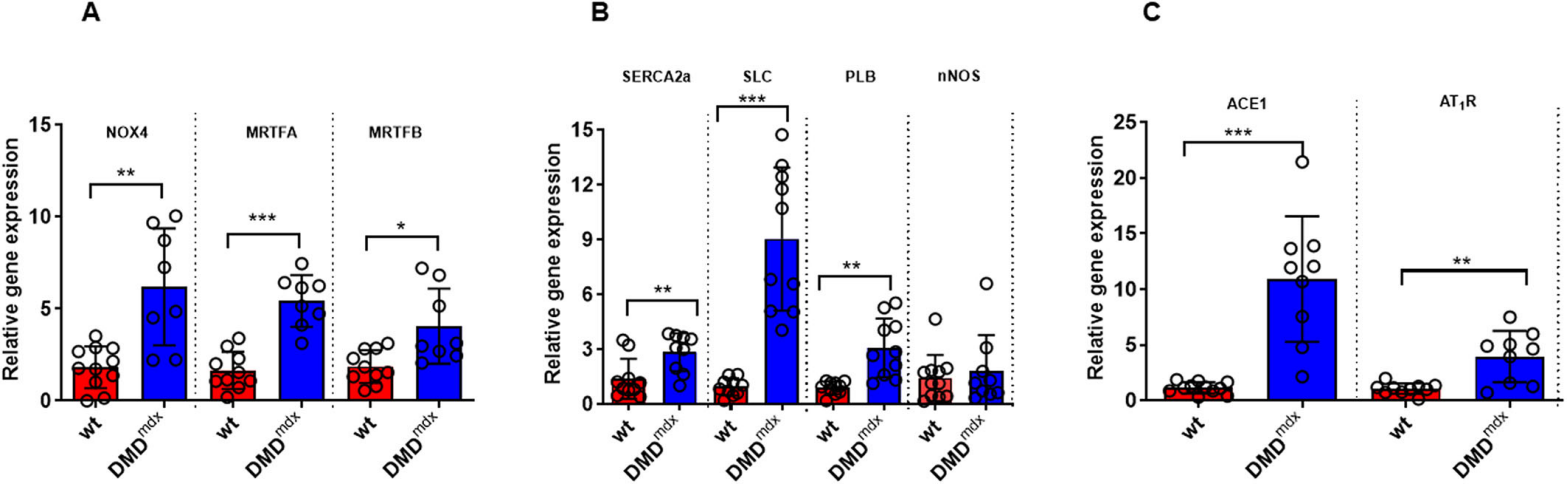
**RT-qPCR data on LV tissue from wt and *Dmd^mdx^* rats at 9 months of age.** NOX4, NADPH oxidase 4; MRTFA, myocardin-related transcription factor A; MRTFB, myocardin-related transcription factor B; SERCA2a, sarcoplasmic reticulum Ca^2+^-ATPase 2a; SLC, sarcolipin; PLB, phospholamban; nNOS, neuronal nitric oxide synthase; ACE1, angiotensin-converting enzyme; AT_1_R, angiotensin II type 1 receptor. Data are expressed as mean±s.d.; *n*=10 wt and *n*=8 *Dmd^mdx^*. *P<0.05, **P<0.01, ***P<0.001, using unpaired Student's *t*-test.

To investigate whether *Dmd^mdx^* rats show altered gene expression associated with cardiomyocyte dysfunction and adverse remodeling, we performed quantitative reverse transcription PCR (RT-qPCR). Upregulation of NADPH oxidase (NOX) 2 and 4 in the myocardium causes cardiac dysfunction and fibrosis (Kuroda et al., 2010). *Nox4* expression was significantly elevated in dystrophic rat hearts compared to wt rat hearts (Fig. 2A). Interestingly, we observed a significant upregulation of myocardin-related transcription factor A and B (*Mrtfa*/*b*) expression in LV tissue samples from *Dmd^mdx^* rats compared to those from wt rats, in association with increases in collagen I and III expression (Fig. S1). In addition, sarcoplasmic reticulum (SR) Ca^2+^-ATPase 2a (*SERCA2a*; also known as *Atp2a2*) mRNA expression was slightly increased in dystrophic compared to wt hearts (Fig. 2B). Next, we investigated phospholamban and sarcolipin, which are key regulators of SERCA activity, and found that, particularly, the expression of sarcolipin was markedly upregulated in *Dmd^mdx^* myocardium (Fig. 2B). The renin–angiotensin–aldosterone system (RAAS) was activated, and angiotensin-converting enzyme (*ACE1*) and angiotensin II type 1 receptor (*AT_1_R*; also known as *Agtr1a*) mRNA levels were significantly increased in dystrophic compared to wt hearts (Fig. 2C). Because dysregulation of neuronal NO synthase (*nNOS*; also known as *Nos1*) and utrophin are supposed to link to cardiac dysfunction (Wehling-Henricks et al., 2005) and compensate for dystrophin deficiency (Delfin et al., 2012), respectively, we measured the expression of both genes in LV tissue samples. There was no difference in *nNOS* mRNA levels (Fig. 2B); however, utrophin mRNA expression was ∼17-fold higher in the *Dmd^mdx^* myocardium compared to wt myocardium (Fig. S2).

### Cardiac fibrosis and inflammation

**Fig. 3.**
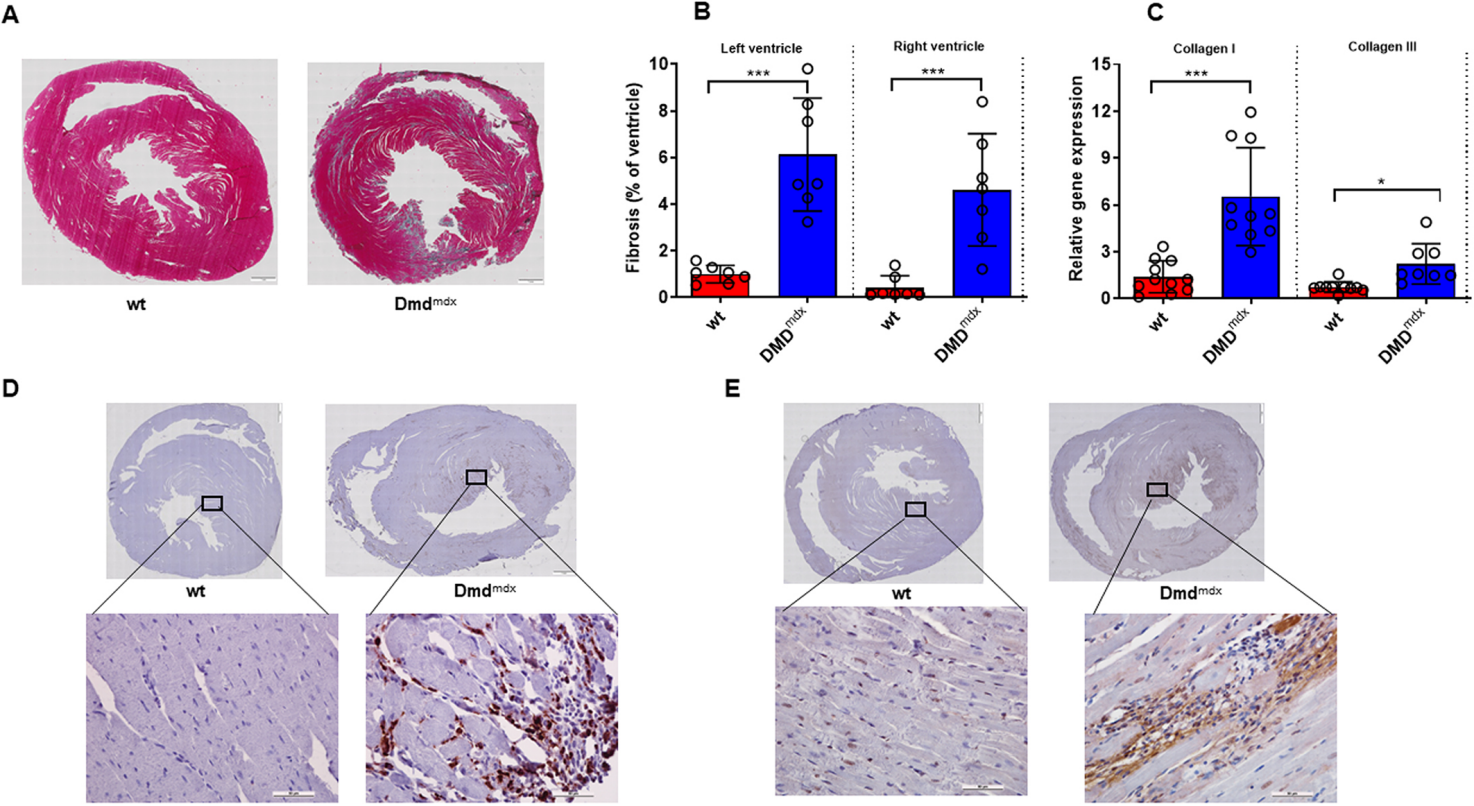
**Histopathological evaluation of cardiac fibrosis and inflammation from wt and *Dmd^mdx^* rats at 9 months of age.** (A) Representative Masson-Goldner staining of LV sections from wt and *Dmd^mdx^* rats at 9 months of age. Blue indicates fibrosis. (B) Quantitative data showing the extent of LV and right ventricular fibrosis. (C) Collagen I and III mRNA expression in myocardium. (D,E) High-magnification photomicrographs show increased levels of CD68^+^ macrophages (D) and tenascin-C (E) expression in *Dmd^mdx^* rat hearts compared to wt rat hearts. Scale bars: 1 mm (overview) and 50 μm (enlarged sections). Data are expressed as mean±s.d.; *n*=7 wt and *n*=7 *Dmd^mdx^* in histology; *n*=11 wt and *n*=10 *Dmd^mdx^* in RT-qPCR. *P<0.05, ***P<0.001, using unpaired Student's *t*-test.

At 9 months of age, we evaluated histological cross sections of the heart at mid-papillary level (Fig. 3A). The results showed that the amount of fibrosis (blue in Fig. 3A) was significantly increased in both the left ventricle and right ventricle in *Dmd^mdx^* compared to wt rats (Fig. 3B). In addition, the RT-qPCR results showed a marked increase in collagen I and III mRNA expression in LV tissue samples of *Dmd^mdx^* rats (Fig. 3C). Furthermore, staining for CD68^+^ macrophages (Fig. 3D) and tenascin-C (TN-C) (Fig. 3E) was enhanced in *Dmd^mdx^* rats compared to wt rats.

### Vascular endothelial function and contractility

**Fig. 4.**
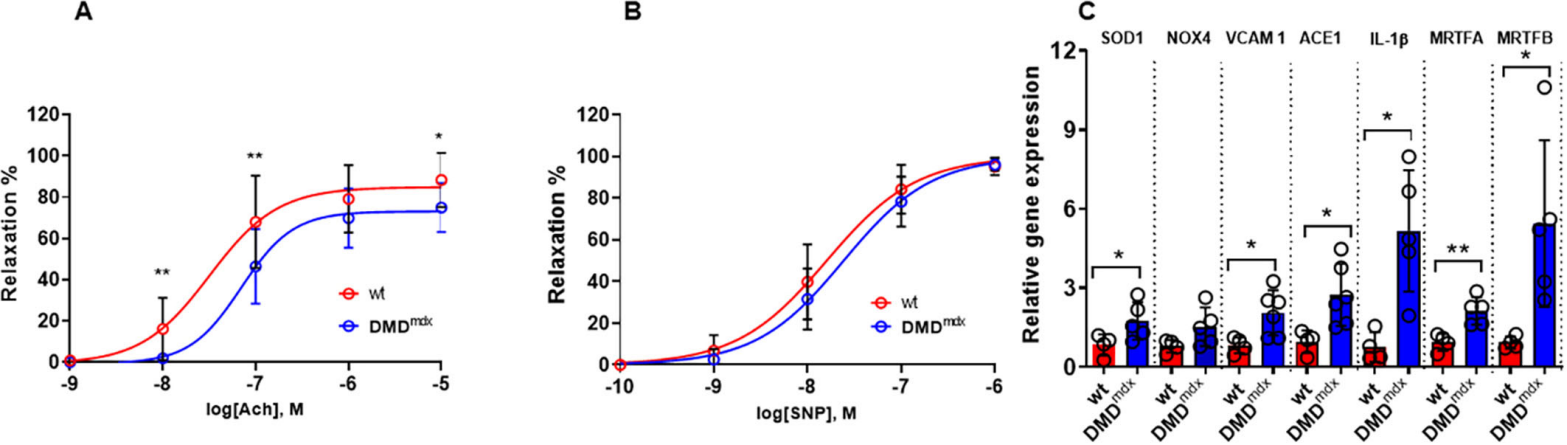
**Vascular reactivity and inflammation in lung endothelial cells of wt and *Dmd^mdx^* rats at 9 months of age.** (A) Endothelium-dependent relaxation in response to acetylcholine (ACh) in aortic rings from wt and *Dmd^mdx^* rats. (B) Relaxations induced by sodium nitroprusside (SNP) in aortic rings from wt and *Dmd^mdx^* rats. Data are expressed as mean±s.d.; *n*=7 wt and *n*=9 *Dmd^mdx^*. *P<0.05, using two-way ANOVA. (C) mRNA expression of *Sod1*, *Nox4*, *Vcam1*, *ACE1*, *Il1b*, *Mrtfa* and *Mrtfb* in wt and dystrophic lung endothelial cells. SOD1, superoxide dismutase; NOX4, NADPH oxidase 4; VCAM1, vascular cell adhesion molecule 1; ACE1, angiotensin-converting enzyme; IL-1β, interleukin 1 beta; MRTFA, myocardin-related transcription factor A; MRTFB, myocardin-related transcription factor B. *n*=4 wt and *n*=5 *Dmd^mdx^* animal. *P<0.05, **P<0.01, using unpaired Student's *t*-test.

Acetylcholine (ACh)-induced vasorelaxation, indicative of endothelium-dependent vasodilation, and sodium nitroprusside (SNP)-induced vasorelaxation, indicative of endothelium-independent vasodilation, were assessed in isolated abdominal aortic segments from *Dmd^mdx^* and wt rats at 9 months of age. In phenylephrine (PE)-preconstricted aortic rings, the relaxation in response to ACh was significantly blunted in *Dmd^mdx^* rats (Fig. 4A), with decreased sensitivity (logEC50, −7.19 versus −7.52 in wt; *P*<0.01), suggesting impaired endothelial-dependent vasorelaxation. In addition, endothelium-independent relaxation induced by SNP showed decreased sensitivity (logEC50, −8.163 versus −7.792 in wt; *P*<0.01; Fig. 4B), but similar maximal response, between the two groups.

### Expression of inflammatory markers in lung ECs

To further elucidate the underlying mechanisms responsible for endothelial dysfunction, CD31^+^ ECs were isolated from the lungs. ECs isolated from *Dmd^mdx^* rat lungs showed higher mRNA expression of pro-inflammatory marker interleukin 1 beta (*Il1b*), and also of mechanical stress-related molecules, such as *Mrtfa* and *Mrtfb* (Fig. 4C). Moreover, superoxide dismutase 1 (*Sod1*) mRNA expression was significantly elevated in ECs isolated from *Dmd^mdx^* rats compared to those from wt rats. In addition, there was a trend toward increased *Nox4* mRNA expression in *Dmd^mdx^* compared to wt ECs (Fig. 4C). Elevated levels of the EC adhesion molecule vascular cell adhesion molecule 1 (*Vcam1*) in ECs from *Dmd^mdx^* rats further indicated an imbalance in vascular function in this animal model of DMD (Fig. 4C).

### ACE activity in kidney, lung and LV tissue samples

**Fig. 5.**
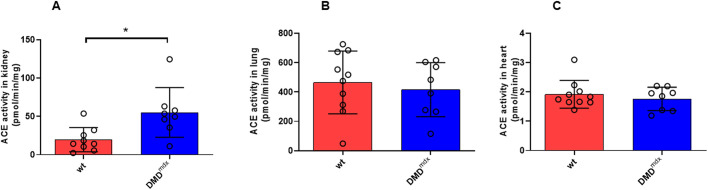
**Angiotensin-converting enzyme (ACE) activity in wt and *Dmd^mdx^* rats.** (A-C) ACE activity in kidney (A), lung (B) and LV (C) tissue samples from wt and *Dmd^mdx^* rats at 9 months of age. Data are expressed as mean±s.d.; *n*=9-10 wt and *n*=8 *Dmd^mdx^*. *P<0.05, using unpaired Student's *t*-test.

To evaluate potential factors contributing to LV dilation and myocardial fibrosis, we assessed ACE activity in kidney, lung and LV tissue samples from *Dmd^mdx^* and wt rats at 9 months of age (Fig. 5A-C). ACE activity was only increased significantly in kidney samples from *Dmd^mdx^* rats in comparison to those from wt rats (Fig. 5A).

### Intracellular Ca^2+^ transients

**Fig. 6.**
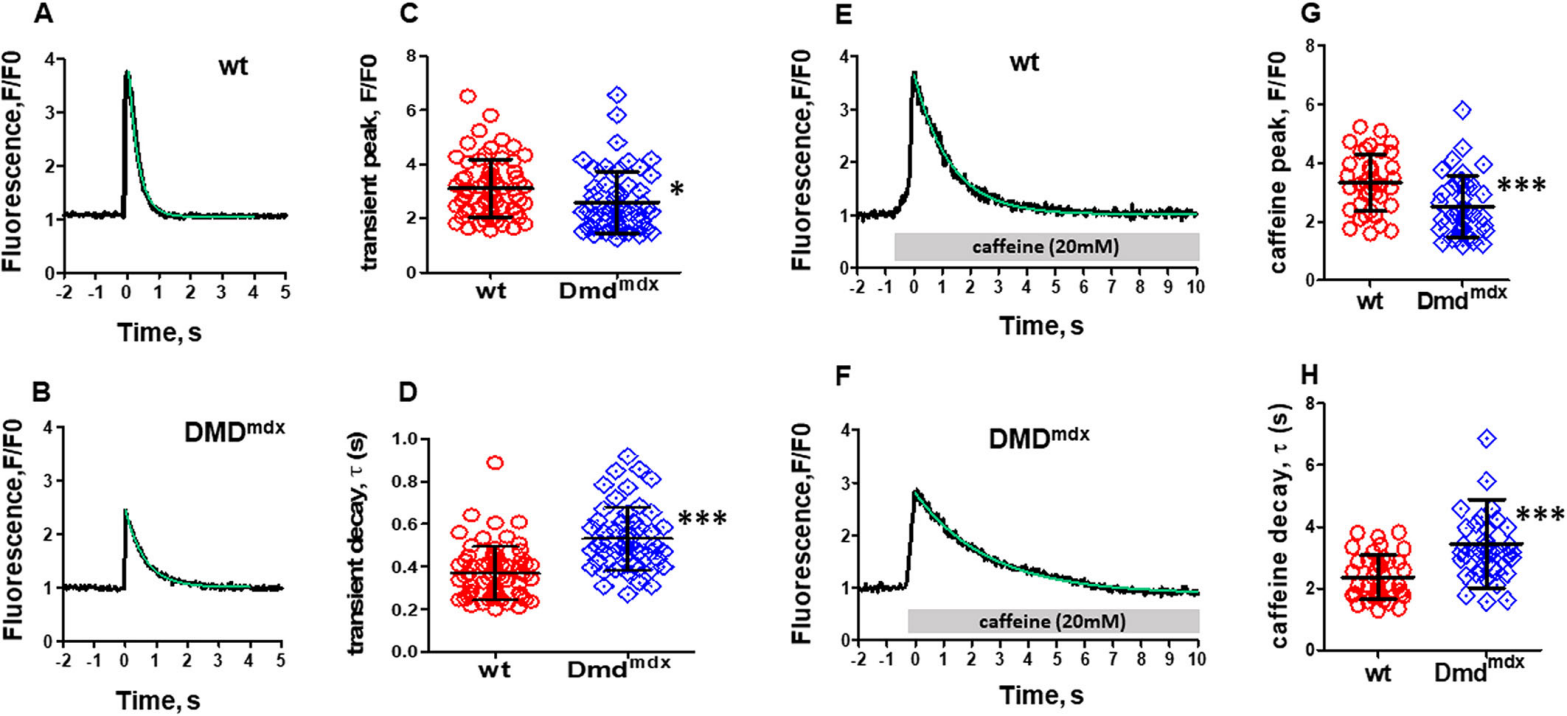
**Electrically stimulated and caffeine-induced intracellular Ca^2+^ transients in wt and *Dmd^mdx^* rat ventricular cardiomyocytes.** (A,B) Representative time courses of Fluo-4 fluorescence reporting rises in cytosolic Ca^2+^ concentration during electrical field stimulation, at 0.2 Hz frequency, in a single wt (A) and *Dmd^mdx^* (B) myocyte. (C) Comparison of mean Ca^2+^ peak fluorescence relative to baseline (F/F0) between wt and *Dmd^mdx^* myocytes. Each data point represents a single cell, and values are expressed as means±s.d. [*n*=67 and *n*=53 for wt (four animals) and *Dmd^mdx^* (four animals) myocytes, respectively]. (D) Comparison of the Ca^2+^ transient decay kinetics in wt and *Dmd^mdx^* cardiomyocytes. The decay of the electrically induced Ca^2+^ signal following the rapid initial rise was fitted with a single exponential function to derive τ-values. (E,F) Time courses of Fluo-4 fluorescence reporting rises in cytosolic Ca^2+^ concentration during application of 20 mM caffeine in a wt (E) and *Dmd^mdx^* (F) myocyte. The gray bars indicate the time period of superfusion with bath solution containing caffeine. (G) Comparison of mean Ca^2+^ peak fluorescence, elicited by caffeine application, relative to baseline (F/F0) between wt and *Dmd^mdx^* myocytes [*n*=40 and *n*=39 for wt (four animals) and *Dmd^mdx^* (four animals) myocytes, respectively]. (H) Comparison of the Ca^2+^ transient decay kinetics (expressed as τ-values) during caffeine application in wt and *Dmd^mdx^* cardiomyocytes. *P<0.05, ***P<0.001, unpaired Student's *t*-test.

Impaired functional properties in the dystrophic heart may arise from abnormalities at the cardiomyocyte level, e.g. impaired cellular Ca^2+^ handling and consequently attenuated contractility. To test this hypothesis, we recorded intracellular Ca^2+^ transients in ventricular cardiomyocytes, which were derived from wt and dystrophic *Dmd^mdx^* rats (Fig. 6A,B). Fig. 6C shows that the amplitude of electrically stimulated Ca^2+^ transients was significantly diminished in *Dmd^mdx^* compared to wt cells. In addition, signal decay in *Dmd^mdx^* myocytes was slowed, as indicated by significantly increased time constant (τ) values (Fig. 6D).

As a next step, we elicited Ca^2+^ transients by means of caffeine (20 mM) application (Fig. 6E-H), and compared peak amplitudes (Fig. 6G) and signal decay (Fig. 6H) between wt and *Dmd^mdx^* myocytes. Similar to what we had observed with electrically induced Ca^2+^ transients, the amplitude of caffeine-induced Ca^2+^ transients was significantly diminished in *Dmd^mdx^* compared to wt cells (Fig. 6G). Further, Ca^2+^ signal decay during continuous caffeine application in *Dmd^mdx^* myocytes was slowed compared to that in wt myocytes (Fig. 6H).

**Fig. 7.**
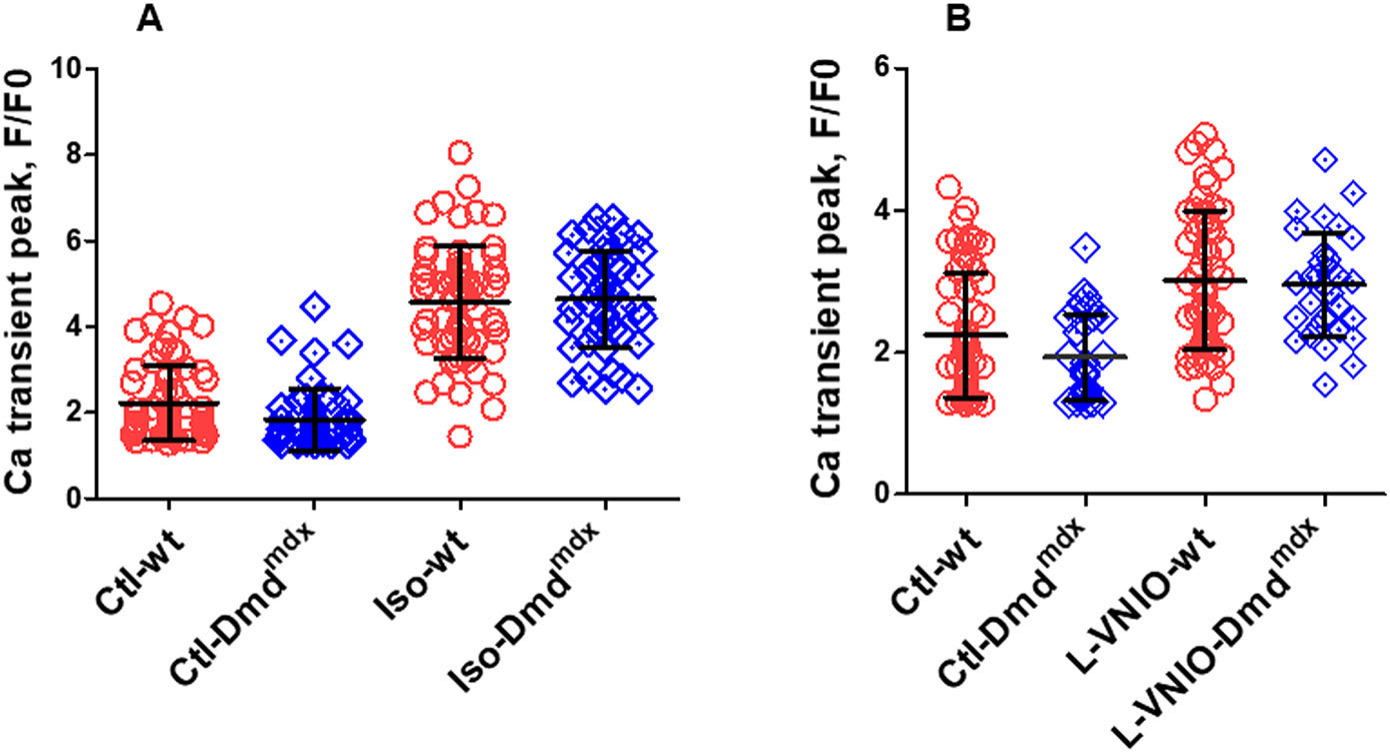
**Regulation of electrically induced (0.2 Hz) Ca^2+^ transients by beta-adrenergic stimulation and inhibition of nNOS signaling in wt and *Dmd^mdx^* ventricular cardiomyocytes.** (A) Comparison of the effect of superfusion with bath solution containing 100 nM isoprenaline on mean Ca^2+^ peak fluorescence relative to baseline (F/F0) in wt and *Dmd^mdx^* myocytes [*n*=63 and *n*=46 for wt (four animals) and *Dmd^mdx^* (four animals) myocytes, respectively]. (B) Comparison of the effect of superfusion with bath solution containing 100 µM of the nNOS inhibitor L-VNIO on mean Ca^2+^ peak fluorescence in wt and *Dmd^mdx^* myocytes [*n*=58 and *n*=31 for wt (three animals) and *Dmd^mdx^* (three animals) myocytes, respectively].

Li et al. (2014) reported that cardiac beta-adrenergic responses were attenuated in dystrophic mdx mice compared to wt mice. Here, we tested whether cardiac beta-adrenergic responsiveness was also impaired in the *Dmd^mdx^* rat model, by comparing isoprenaline effects on electrically stimulated Ca^2+^ transients in wt and *Dmd^mdx^* myocytes. Fig. 7A shows that external application of 100 nM isoprenaline led to a prominent increase in Ca^2+^ transient amplitude both in wt and *Dmd^mdx^* cells. Similar transient peak amplitudes were reached under isoprenaline stimulation in both cell types.

Nitric oxide (NO), generated by nNOS activity, has an inhibitory effect and significantly diminishes Ca^2+^ transients (Burger et al., 2009; Sears et al., 2003). Here, we tested the stimulatory effects of the selective cell permeable nNOS inhibitor vinyl-L-NIO (hydrochloride) (L-VNIO; Cayman Chemical, CAY-80330-5) on Ca^2+^ transients recorded from wt and *Dmd^mdx^* myocytes. We found that external application of 100 µM L-VNIO increased the transient peak amplitudes in wt and dystrophin-deficient cells (Fig. 7B). As described above for isoprenaline stimulation, L-VNIO application also resulted in similar Ca^2+^ transient peak amplitude values in both cell types.

### Ca_v_1.2 protein expression and L-type Ca^2+^ channel currents

**Fig. 8.**
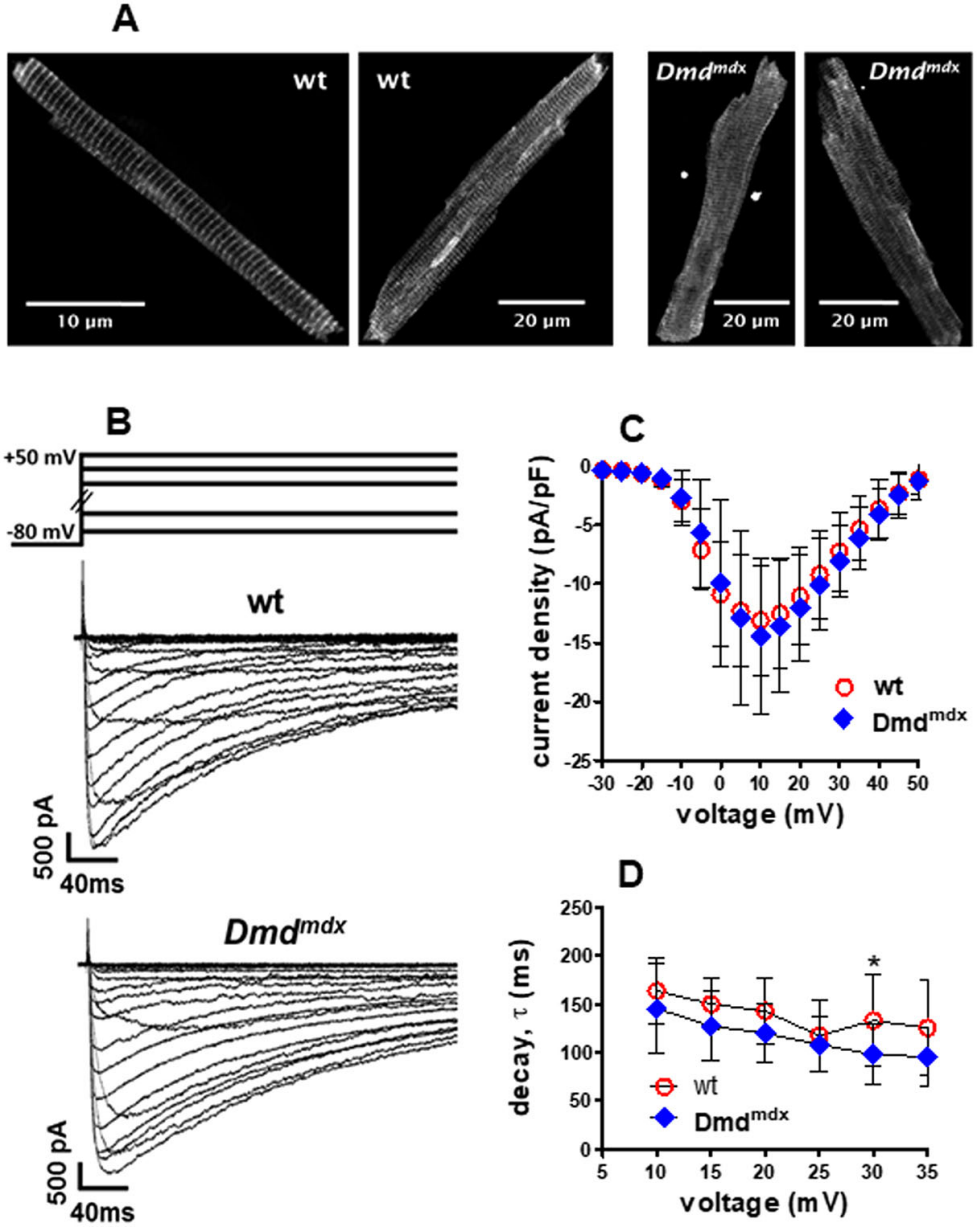
**L-type Ca^2+^ channel expression and function in wt and *Dmd^mdx^* ventricular cardiomyocytes.** (A) Cav1.2 Ca^2+^ channel expression and localization in single isolated wt and *Dmd^mdx^* ventricular cardiomyocytes. Two typical examples of Cav1.2 immunostaining in wt and *Dmd^mdx^* myocytes are shown. (B-D) L-type Ca^2+^ channel properties in wt and *Dmd^mdx^* ventricular cardiomyocytes. (B) Typical examples of original traces of barium currents through Ca^2+^ channels in a wt and *Dmd^mdx^* myocyte elicited by the pulse protocol displayed on top. (C) The inward current peaks at the respective applied membrane potentials, derived from a series of such experiments, were divided by the cell capacitance to obtain the current density-voltage relationship. Values are expressed as means±s.d. [*n*=10 and *n*=13 for wt (four animals) and *Dmd^mdx^* (four animals) myocytes, respectively]. (D) Barium current decay kinetics (expressed as τ-values derived from single exponential fits of the current decay after channel activation) at different membrane potentials were compared between wt and *Dmd^mdx^* myocytes. *P=0.046, using unpaired Student's *t*-test.

To study potential abnormalities in Ca_v_1.2 Ca^2+^ channel expression and localization in *Dmd^mdx^* ventricular cardiomyocytes, we performed immunofluorescence studies using an antibody specific for the Ca_v_1.2 alpha1 subunit (also known as CACNA1C). Fig. 8A compares typical examples of Ca_v_1.2 immunostaining in wt and *Dmd^mdx^* myocytes. Cross-striations, representing T-tubular localization of Ca_v_1.2 Ca^2+^ channels, can be observed in wt and dystrophin-deficient cells. Similar staining patterns, with comparable signal intensities under identical excitation conditions and microscope settings, were obtained in all the studied wt and *Dmd^mdx^* myocytes, which originated from two wt and two dystrophic rats, respectively.

In a final set of experiments, we tested potential abnormalities in Ca^2+^ channel functional properties. Therefore, we recorded currents through L-type Ca^2+^ channels in wt and *Dmd^mdx^* cardiomyocytes. Fig. 8B-D show that the currents in wt and dystrophin-deficient cells were basically similar. Thus, first, no significant difference existed between the current density-voltage relationships of wt and *Dmd^mdx^* myocytes (Fig. 8C). Second, current decay after channel activation at various membrane potentials, representing the kinetics of macroscopic Ca^2+^ channel inactivation, was similar in wt and dystrophin-deficient cells (Fig. 8D). A statistically significant difference (a faster decay in *Dmd^mdx^* versus wt; *P=0.046; Fig. 8D) only existed at a potential of +30 mV.

## DISCUSSION

Here, for the first time, we provide a detailed characterization of the cardiovascular abnormalities of *Dmd^mdx^* rats (Larcher et al., 2014). Our study also reveals potential signaling mechanisms that may play a role in cardiomyocyte and EC dysfunction, as well as in adverse LV remodeling, in the dystrophic heart. In summary, we found that the hearts of *Dmd^mdx^* rats show significant and progressive diastolic and systolic dysfunction, as well as cardiac fibrosis and inflammation, consistent with the development of a pronounced dilated cardiomyopathy. Moreover, in *Dmd^mdx^* rats, vascular endothelial function is impaired, and Ca^2+^ handling in *Dmd^mdx^* cardiomyocytes is abnormal.

### Comparison of cardiac function between *Dmd^mdx^* rats and DMD patients

In contrast to Larcher et al. (2014), in the present study, we went further and investigated LV remodeling and cardiac function beyond the age of 3 months; namely, after 7 and 9 months. Our data at 3 months confirmed the results of Larcher et al. (2014), but we were only able to demonstrate a trend toward diastolic dysfunction. However, we found a significant reduction in EF at an early stage (3 months). E/E′ ratio, as a surrogate marker of diastolic function, was significantly increased in *Dmd^mdx^* rats compared to wt rats at 7 and 9 months, confirming the diastolic dysfunction in *Dmd^mdx^* rats. Echocardiography data were supported by invasive LV pressure measurements at sacrifice (9 months). Accordingly, *Dmd^mdx^* rats showed lower LV maximum pressure, dP/dt_max_ and dP/dt_min_. In addition, there was a significant difference in LVEDP between the two groups.

The rapid and progressive dilatation and contractile dysfunction in *Dmd^mdx^* rats is in alignment with the cardiac complications in DMD patients, as cardiac involvement becomes more prominent with age (Nigro et al., 1990). It has been also shown that DMD and Becker muscular dystrophy (BMD) patients express abnormally high levels of utrophin (Taylor et al., 1997), similar to what we observed in LV tissue samples of *Dmd^mdx^* compared to wt rats (Fig. S2). The level of this protein is indicating compensatory mechanisms, as utrophin can partially substitute dystrophin. Notably, pulmonary hypertension is a typical malformation of patients affected by DMD owing to respiratory failure. This underlines the potential of *Dmd^mdx^* rats to study the dystrophic disease phenotype (Yotsukura et al., 1991). Of importance, LV function in patients with DMD declined over time, independent of non-invasive positive pressure ventilation use (James et al., 2020).

### Comparison of cardiac morphology, inflammation and neurohumoral activation between *Dmd^mdx^* rats and DMD patients

Dilated cardiomyopathy is typically observed in DMD patients and characterized by LV dilatation and excessive myocardial fibrosis (Olivieri et al., 2016). In line with that, *Dmd^mdx^* rats exhibited an enlargement of LVESD (at 3, 7 and 9 months) and LVEDD (at 3, 7 and 9 months) compared to wt animals. Previous studies demonstrated that an increase in LV dilatation and concomitant reduced systolic function increase the risk of ventricular arrhythmia (e.g. Finsterer et al., 2010), confirming the suitability of the *Dmd^mdx^* rat model for studying the molecular and cellular mechanisms of electrophysiological abnormalities in DMD.

Patients affected by DMD develop a progressive dilated cardiomyopathy, characterized by inflammatory cell infiltration, followed by necrosis and excessive replacement with cardiac fibrosis (Olivieri et al., 2016). The fibrotic region gradually becomes thinner and loses contractility, resulting in dilated cardiomyopathy (Kaspar et al., 2009). More recently, Ouisse et al. (2019) demonstrated that CD68^+^ positive cell staining was markedly increased in cardiac tissue of *Dmd^mdx^* rats at the age of 12 weeks. In line with that, our study confirms a large amount of fibrosis and CD68^+^ macrophage infiltration in both left and right ventricles in *Dmd^mdx^* rats at the age of 9 months. Collectively, these data suggest that targeting inflammation and cardiac fibrosis are potential approaches to delay and limit cardiac dysfunction in DMD patients.

Accordingly, a recent randomized clinical trial has demonstrated that drugs inhibiting the RAAS, including ACE inhibitors, significantly reduce the progression of myocardial fibrosis in patients with DMD or BMD (Silva et al., 2017), suggesting a deleterious role of ACE in DMD-related myocardial fibrosis. Therefore, we measured ACE activity in various organs and *AT_1_R* expression in LV tissue. We first demonstrated that ACE activity was significantly increased in kidney tissue from *Dmd^mdx^* rats, but was not altered in lungs and LV tissue samples. However, *AT_1_R* expression was significantly increased in LV tissue, which may support the benefit of using ACE inhibitors or AT_1_R blockers to delay cardiac dysfunction and concomitant fibrosis in DMD patients. Besides, ACE produces the potent vasoconstrictor angiotensin II, which is known to be a key mediator in the development of pulmonary hypertension, vascular remodeling and endothelial dysfunction (Cohn, 2000). Additionally, a mechanistic link between angiotensin II and TN-C has recently been demonstrated (Santer et al., 2020). Further, TN-C knockout mice develop less LV dilatation and fibrosis under chronic pressure overload condition than wt mice (Podesser et al., 2018). In line with that, upregulation of TN-C in serum or cardiac tissue predicted worse outcome in patients with myocardial infarction (Sato et al., 2006) and dilated cardiomyopathy (Yokokawa et al., 2016). In the present study, we were able to demonstrate, for the first time, that cardiac TN-C expression was markedly increased in *Dmd^mdx^* compared to wt rats, suggesting its pathophysiological role in LV dilation and fibrosis. Of importance, a previous clinical study reported positive interaction between LV dilatation and TN-C levels in DMD and Emery-Dreifuss muscular dystrophy patients (Niebroj-Dobosz et al., 2011).

Besides the evidence-based pro-fibrotic effects of RAAS upregulation in DMD, our study results suggest that MRTFA may be a novel potential mediator, as well as target, in cardiac fibrosis in DMD. Previous studies demonstrated the role of MRTFA in cardiac fibrosis (Osmanagic-Myers et al., 2019; Sharma et al., 2017). Accordingly, *Mrtfa* upregulation in LV tissue samples from *Dmd^mdx^* rats was associated and correlated with collagen I and III expression (Fig. S1).

### Comparison of vascular function between *Dmd^mdx^* rats and DMD patients

Little is known about vascular function in DMD; however, the role of the vasculature can no longer be ignored in light of the mounting evidence for its importance in the pathogenic process (Ennen et al., 2013). Therefore, we investigated vascular function in *Dmd^mdx^* rats. We compared endothelium-dependent and -independent vasodilator responsiveness in aorta segments from *Dmd^mdx^* and wt animals. Myograph data indicated impaired endothelium-dependent relaxation. To shed some light on the molecular mechanisms by which the DMD phenotype promotes endothelial dysfunction, we examined markers of inflammation, mechanical and oxidative stress in isolated lung ECs. We found increased expression of oxidative and mechanical stress markers, such as *Nox4* and *Mrtfa*, as well as higher expression of *Vcam1*, in *Dmd^mdx^* compared to wt ECs. There are limited studies that have investigated vasorelaxation capacity in DMD patients. Among those, Miike et al. (1987) demonstrated EC injury in DMD patients. Other investigators found that improvement of vascular function by ACE (Russo et al., 2018) or phosphodiesterase type 5 (Nelson et al., 2014) inhibitors brings along benefit in DMD patients, underlining the pathophysiological importance and potential target of vascular endothelium in DMD-associated cardiovascular abnormalities.

### Correlation between *Dmd^mdx^* rat ‘organ’ and ‘cellular’ abnormalities

Abnormal Ca^2+^ transients are a pivotal feature of failing hearts: typically, Ca^2+^ transients in ventricular cardiomyocytes from heart failure patients are smaller, and their duration is prolonged (Balke and Shorofsky, 1998; Benitah et al., 2003; Beuckelmann et al., 1992). Importantly, these alterations at the cellular (cardiomyocyte) level are causative for impaired cardiac function (Balke and Shorofsky, 1998; Gambardella et al., 2018; Pieske et al., 1995). Thus, reduced Ca^2+^ release from the SR impairs cardiomyocyte contractility, and consequently systolic function, whereas diminished Ca^2+^ removal from the cytosol after SR Ca^2+^ release increases diastolic dysfunction (Balke and Shorofsky, 1998; Gambardella et al., 2018; Hoshijima et al., 2006). Based on these facts, we propose that the functional impairments of the heart in *Dmd^mdx^* rats reported herein can at least partly be explained by the altered Ca^2+^ transient properties in *Dmd^mdx^* ventricular cardiomyocytes. In particular, the reduced Ca^2+^ transient amplitude in dystrophin-deficient myocytes may contribute to impaired systolic function in the *Dmd^mdx^* heart, and the slowed transient decay in *Dmd^mdx^* cells is consistent with diastolic dysfunction in the dystrophic heart. Finally, it should also be mentioned that abnormal Ca^2+^ transients in dystrophin-deficient cardiomyocytes predispose the dystrophic heart to cardiac arrhythmias (Fauconnier et al., 2010).

### Potential mechanisms underlying abnormal Ca^2+^ handling in *Dmd^mdx^* cardiomyocytes

Impaired Ca^2+^ transients, but at the same time normal L-type Ca^2+^ channel expression and functional properties in *Dmd^mdx^* cardiomyocytes (see above), exclude Ca_v_1.2 Ca^2+^ channel dysfunction as a triggering mechanism for abnormal Ca^2+^ release. Decreased electrically evoked Ca^2+^ transient amplitudes, suggesting reduced Ca^2+^ release from the SR via ryanodine receptors, in dystrophin-deficient myocytes may alternatively arise from disturbed ryanodine receptor function in the SR membrane and/or a diminished SR Ca^2+^ load. Our caffeine experiments provide support for the latter mechanism – reduced Ca^2+^ content of the SR, potentially caused by leaky ryanodine receptors, as in dystrophin-deficient murine mdx cardiomyocytes (Fauconnier et al., 2010; Gonzalez et al., 2014; Shirokova and Niggli, 2013). Thus, caffeine-induced Ca^2+^ transient amplitude, a measure for SR Ca^2+^ load (Williams and Allen, 2007), was significantly decreased in *Dmd^mdx^* compared to wt cardiomyocytes. Finally, our data also exclude impaired Ca^2+^ channel inactivation as cause for the prolonged electrically evoked Ca^2+^ transient duration observed in *Dmd^mdx^* cardiomyocytes. Consequently, removal of Ca^2+^ from the cytosol after release, reflected by decay of the Ca^2+^ signal, must be attenuated by a direct mechanism such as impaired SERCA function. In accordance, SERCA activity was shown to be reduced in dystrophin-deficient mouse hearts (Voit et al., 2017; Williams and Allen, 2007). An abnormally decreased rate of Ca^2+^ uptake by the SR was also found in ventricular cardiomyocytes from patients with dilated cardiomyopathy (Beuckelmann et al., 1995).

The reason for reduced SERCA activity in dystrophic rat cardiomyocytes remains unknown. Our RT-qPCR studies point to a role of sarcolipin. This protein is a potent SERCA inhibitor (Voit et al., 2017), and its expression is massively upregulated in *Dmd^mdx^* rat hearts (Fig. 2B). This finding is in line with abnormally elevated sarcolipin levels in the muscle of DMD patients and mouse models of the disease (Voit et al., 2017). Most interestingly in this context, in recent studies, both a single systemic delivery of SERCA2a with adeno-associated virus (Wasala et al., 2020) and sarcolipin deletion (Voit et al., 2017) improved Ca^2+^ recycling and provided considerable benefits in mouse models of DMD.

Finally, our study additionally suggests that, at the cardiomyocyte level, the regulation of Ca^2+^ handling via the beta-adrenergic pathway and via nNOS activity is not impaired by dystrophin deficiency.

### Comparison of cytosolic Ca^2+^ transient properties in rat *Dmd^mdx^* and human DMD cardiomyocytes

Here, we report decreased electrically evoked Ca^2+^ transient amplitudes and a prolonged Ca^2+^ transient duration (slowed signal decay) in dystrophin-deficient *Dmd^mdx^* compared to wt cardiomyocytes. The latter abnormality was also observed in human induced pluripotent stem cell (iPSC)-derived cardiomyocytes from a DMD patient (Guan et al., 2014). Also, cardiomyocytes isolated from the ventricular myocardium of dilated cardiomyopathy patients showed comparable abnormalities in electrically evoked Ca^2+^ transients, as we observed in *Dmd^mdx^* myocytes: a decreased Ca^2+^ transient amplitude and a slowed transient decay compared to myocytes derived from healthy donor ventricles (Beuckelmann et al., 1992, 1995). Finally, our finding of decreased caffeine-evoked Ca^2+^ transient amplitudes in *Dmd^mdx^* compared to wt rat myocytes accords with abnormally small caffeine-induced Ca^2+^ transients in myocytes derived from patients with terminal heart failure owing to dilated cardiomyopathy or ischemic heart disease (Lindner et al., 1998). Together, these comparisons suggest that, as far as Ca^2+^ transient properties in ventricular cardiomyocytes are concerned, the *Dmd^mdx^* rat model well resembles the situation observed in diseased (DMD, dilated cardiomyopathy) human myocytes.

### Comparison of L-type Ca^2+^ channel properties in rat *Dmd^mdx^* and human DMD cardiomyocytes

Dystrophin-deficient *Dmd^mdx^* rat myocytes show Ca^2+^ channel properties very similar to those of wt cells. In line with these rat model data, iPSC-derived cardiomyocytes from DMD patients had similar Ca^2+^ current densities to myocytes derived from a healthy control individual (E. Jimenez Vazquez, University of Michigan, personal communication). Further, cardiomyocytes isolated from the ventricular myocardium of dilated cardiomyopathy patients had comparable Ca^2+^ current densities to myocytes derived from healthy donor ventricles (Beuckelmann et al., 1992). This suggests that, regarding Ca^2+^ current properties in ventricular cardiomyocytes, the *Dmd^mdx^* rat model resembles the situation observed in human disease.

### Limitations

Certain limitations of the study have to be acknowledged. First, we only measured cardiac fibrosis, vascular function and cardiomyocyte properties at a defined age of 9 months. We therefore do not provide information about disease development. Second, we only measured markers related to cardiac remodeling, Ca^2+^ handling in cardiomyocytes and oxidative stress in ECs at mRNA level. Validation at protein level should be part of future studies.

### Conclusion

In conclusion, we were able to show that *Dmd^mdx^* rats represent a promising small-animal model to elucidate mechanisms of cardiomyopathy development in the dystrophic heart. Accordingly, *Dmd^mdx^* rats show significantly impaired LV hemodynamic function and adverse remodeling with concomitant cardiac fibrosis and inflammation. Furthermore, in *Dmd^mdx^* rats, vascular endothelial function is impaired, which may be related to inflammation and oxidative stress marker upregulation. At the cellular level, Ca^2+^ handling in *Dmd^mdx^* cardiomyocytes is abnormal. Collectively, in contrast to the classical mdx mouse model, the cardiovascular phenotype of *Dmd^mdx^* rats is developing quickly, strong and very similar to that observed in DMD patients. We therefore believe that *Dmd^mdx^* rats represent a suitable small-animal model to test novel therapies aiming to combat cardiovascular complications in DMD and other forms of dilated cardiomyopathies.

## MATERIALS AND METHODS

### Animals

Male *Dmd^mdx^* (*n*=14) and wt littermate control (*n*=15) Sprague Dawley rats were from INSERM-CRTI UMR 1064. Genotyping of the rats was performed using standard PCR assay as described previously (Larcher et al., 2014). The experimental protocol was approved by the regional Ethics Committee for Laboratory Animal Experiments at the Medical University of Vienna and the Austrian Ministry of Science Research and Economy (BMWFW-66.009/0175-WF/V/3b/2015). All procedures conform to the guidelines from Animal Research: Reporting of *In Vivo* Experiments (ARRIVE) and Directive 2010/63/EU of the European Parliament on the protection of animals used for scientific purposes.

### Transthoracic echocardiography assessment

Transthoracic echocardiography was performed as described previously (Pilz et al., 2019). Briefly, rats were anesthetized (isoflurane 2-3%) and echocardiography was performed using a Vivid7 system (GE Healthcare, USA) equipped with an 11.5 MHz 10S sector transducer. LV EF, LVEDD and LVESD were evaluated at midpapillary short-axis view. Tissue Doppler of the septal mitral annulus as well as the mitral flow were obtained in a four-chamber view to evaluate LV filling pressure. From the transaortic parasternal short axis view, pulsed wave Doppler flow tracings from the pulmonary artery were obtained, and pulmonary artery acceleration time (PAAT) was measured as the time from onset of the flow to the peak flow velocity. mPAP was then calculated using a regression equation validated in rats: mPAP=58.7−(1.21×PAAT) (Urboniene et al., 2010).

### Assessment of LV hemodynamic parameters

LV hemodynamic parameters were invasively measured. Rats (at 9 months of age) were anesthetized by intraperitoneal injection of a mixture of xylazine (4 mg/kg; Bayer, Germany) and ketamine (100 mg/kg; Dr E. Gräub AG, Switzerland), intubated and ventilated. The chest was opened and a microtip catheter (SPR-409, 2F, Millar Instruments, Houston, TX, USA) was gently inserted into the LV chamber. Hemodynamic parameters such as LV systolic (LVSP), LVEDP and heart rate were continuously recorded using LabChart (v7.3.2) and PowerLab System (8/30; both ADInstruments, Spechbach, Germany). Thereafter, the heart and lungs were removed and rinsed in ice-cold saline, before major blood vessels and connective tissue were removed, the heart blotted dry and weighed, and the heart or lung weight/body weight ratio calculated.

### Histological and immunohistochemical analyses

Formalin-fixed paraffin-embedded tissue sections were Hematoxylin and Eosin stained. The extent of interstitial fibrosis in cardiac muscle sections was visualized by Masson-Goldner staining (Masson-Goldner staining kit, Sigma-Aldrich/Merck, Darmstadt, Germany). Images were acquired by microscopy (VS120 Virtual Slide Microscope System, Olympus, Tokyo, Japan) and captured by a digital camera (AVT PIKE F-505C VC 50, Allied Vision Technologies, Stadtroda, Germany). To assess myocardial interstitial fibrosis, the LV and right ventricular (RV) area was estimated using a slice obtained from the central part of the myocardium at mid-papillary level. The percentage of interstitial fibrosis was acquired with Adobe Photoshop Element (version 14.1) based on the following equation: % fibrosis (RV or LV)=fibrotic area/ fibrotic area+non-fibrotic area (LV or RV).

For immunohistochemistry, deparaffinized cardiac tissue sections were incubated with antibodies against CD68 (1:100; mouse monoclonal, ED1, Abcam, Cambridge, MA, USA) to evaluate the density of tissue macrophages, or against TN-C (1:25; rabbit polyclonal, Chemicon, Temecula, CA, USA) as described previously (Pilz et al., 2019). Briefly, primary antibodies were detected with appropriate biotinylated secondary antibody (Vector Laboratories, Burlingame, CA, USA) and horseradish peroxidase (HRP)-conjugated streptavidin (Dako, Glostrup, Denmark), developed with 3,3′-diaminobenzidine (DAB) (Vector Laboratories), counterstained with Hematoxylin, dehydrated and mounted in DPX (Merck, Darmstadt, Germany). Digitalized images were generated with an Eclipse 80i (Nikon, Tokyo, Japan) microscope.

### Assessment of vascular reactivity in isolated aortic rings

Vascular function was assessed in isolated aortic rings as described previously (Osmanagic-Myers et al., 2019). Briefly, rat abdominal aorta (at the age of 9 months) was dissected and placed into cold and oxygenated (5% CO_2_ and 95% O_2_) Krebs buffer containing 119 mmol/l NaCl, 4.7 mmol/l KCl, 2.5 mmol/l CaCl_2_, 1.17 mmol/l MgSO_4_, 20 mmol/l NaHCO_3_, 1.18 mmol/l KH_2_PO_4_, 0.027 mmol/l EDTA and 10.5 mmol/l glucose. The segments of the aorta were gently perfused to remove all the remaining blood from the lumen and cleaned of the connective tissue around the vessel. Aortic rings (2-3 mm) were mounted onto a multichamber isometric myograph system (Model 620 M, Danish Myo Technology, Aarhus, Denmark). The organ chambers of the myograph were filled with heated (37°C) and oxygenated Krebs solution, and the individual chambers were further heated and bubbled during the whole procedure. Segments were allowed to equilibrate for 45 min and resting tension was continuously adjusted during this period as described previously (Szekeres et al., 2015). Reference contractions were elicited by hyperkalemic (124 mM, KCl) solution. Precontraction of the segments was performed by PE (1 nM-10 µM; Sigma-Aldrich). Endothelial-dependent and -independent relaxation was tested by ACh (1 nM-10 µM; a nitric oxide-dependent vasodilator; Sigma-Aldrich) and SNP (0.1 nM-1 µM; a nitric oxide-independent vasodilator; Merck), respectively. The data were continuously recorded using the software program LabChart Pro (ADInstruments).

### Isolation of lung ECs

Lungs were removed and placed into ice-cold PBS before being finely minced. The fragments were collected in pre-warmed (37°C) 2 mg/ml Collagenase-PBS solution (Collagenase Type IV from Gibco) and incubated for 45 min at 37°C. The mixture was passed through a 70-µm cell strainer. The red blood cells were eliminated by red blood cell lysis buffer (Roche). Mouse anti-rat CD31 antibody (BD Pharmingen) was incubated with magnetic beads coated with pan anti-mouse IgG (Dynabeads™, Invitrogen) on a rotator plate at 4°C overnight (100 µl magnetic beads and 10 µl anti-rat CD31 antibody in 4 ml 0.1% PBS-BSA buffer is calculated for one rat lung). The rat lung cell suspension was incubated together with the magnetic beads on a rotator plate for 20 min at room temperature and then the CD31^+^ cells were separated by a magnetic particle concentrator (Dynabeads™ MPC™-1 Magnet). After the CD31^+^ cells were purified by three subsequent washing and separation steps by the magnetic particle concentrator, cell lysis buffer was added and total RNA was isolated from the lung ECs.

### ACE activity measurement

ACE activity in heart, lung and kidney tissue samples was measured as originally described by Carmona et al. (2006) and modified by Fagyas et al. (2014). Briefly, tissue samples were weighed and a proportional amount of 100 mM tris(hydroxymethyl)aminomethane hydrochloride (TRIS) buffer (pH 7.0) was added then homogenized. The tissue homogenates were centrifuged at 15,000 ***g*** for 5 min, and the protein concentration of the supernatant was determined by a Pierce^TM^ BCA Protein Assay Kit (Thermo Fisher Scientific) using a TECAN (SparkControl Magellan V2.2) plate reader. ACE activity was determined with an artificial substrate (Abz-FRK(Dnp)P-OH (synthesized by Peptide 2.0, Chantilly, VA, USA) in a reaction mixture containing 6 µl of 1 mg/ml tissue homogenates in 35-fold dilution in 100 mM TRIS buffer, 50 mM NaCl, 10 µM ZnCl_2._ Measurements were performed in 96-well plates (Greiner-Bio One) at 37°C. The fluorescence intensity change was detected by a TECAN (SparkControl Magellan V2.2) plate reader (excitation, 340 nm; emission, 405 nm). The changes in fluorescence intensity were detected in kinetic loops, at 1-min intervals for at least 30 min and the intensity values were plotted as a function of reaction time. The fluorescence intensity values were fitted by a linear regression (GraphPad Software, San Diego, CA, USA), and the fit with the data was accepted only when r^2^ was >0.9. ACE activity was calculated by the following equation: activity=(*S/k*)×*D/P*, where *S* is the rate of the increase in fluorescence intensity (1/min), *k* is the change in fluorescence intensity during the complete cleavage of 1 pmol Abz-FRK(Dnp)P-OH substrate, *D* is the dilution of the sample, and *P* is the mg/ml protein concentration; 1 unit (U) means 1 pmol substrate cleavage in 1 min by 1 mg of protein.

### RT-qPCR

Total RNA and miRNA were extracted and isolated using a miRNeasy Mini Kit (Qiagen, Hilden, Germany) from rat LV tissue and lung EC cell suspension according to the protocol provided. Briefly, QIAzol buffer was added to each tube containing either 50 mg myocardium for tissue homogenization using a tissue rupture (Qiagen) or lung EC cell suspension. Chloroform was added, and, after centrifugation, a volume of absolute ethanol was added to the upper phase and transferred to a miRNeasy Mini spin column. After several washing steps, total RNA was obtained after adding 30 μl nuclease-free water. RNA concentration was measured using NanoQuant plate^TM^ and TECAN plate reader (SparkControl Magellan V2.2). cDNA was prepared using a QuantiTect reverse transcription kit (Qiagen), according to the manufacturer's instructions. After cDNA preparation, quantitative PCR was performed using a QuantiTect SYBR Green PCR kit (Qiagen). Samples were analyzed in duplicate using ROTOR-Gene Q (Qiagen). Relative gene expression (listed in Table S1) was calculated by 2^−ΔΔCt^ method.

### Isolation of ventricular cardiomyocytes

Male wt and *Dmd^mdx^* rats (*n*=4 wt and *n*=4 *Dmd^mdx^* animals) at the age of 9 months were killed by cervical dislocation. Cardiomyocytes were isolated from the ventricles of their hearts using a Langendorff setup according to the myocyte isolation procedure from mice described in our previous work (Koenig et al., 2011).

### Intracellular Ca^2+^ transient measurements

Ca^2+^ transients were recorded from isolated rat ventricular wt and *Dmd^mdx^* cardiomyocytes at room temperature following the protocol described in detail in our recently published study (Rubi et al., 2018). In brief, myocytes pre-loaded with the cell membrane-permeable Ca^2+^-sensitive fluorescent dye Fluo-4 AM (Thermo Fisher Scientific, Vienna, Austria) were bathed in an extracellular solution containing 140 mmol/l NaCl, 4 mmol/l KCl, 2 mmol/l CaCl_2_, 2 mmol/l MgCl_2_, 5 mmol/l HEPES, 5 mmol/l glucose, pH adjusted to 7.4 with NaOH. Electrical stimulation via platinum electrodes in the bath was performed at 0.2 Hz in order to elicit Ca^2+^ transients. To elicit caffeine-induced SR Ca^2+^ release, bath solution containing 20 mmol/l caffeine was applied via an OctaFlow II perfusion system (ALA Scientific Instruments, Westbury, NY, USA). Dye fluorescence signals were acquired by means of a confocal microscope system (Nikon A1R+). Fluorescence peaks upon stimulation with single electrical pulses or with caffeine were evaluated relative to baseline fluorescence prior to stimulation (F0). To evaluate the duration of the elicited Ca^2+^ transients, a single exponential function was fitted to the decaying fluorescence to obtain respective time constants (τ-values).

### Ca_v_1.2 immunostaining

Isolated ventricular cardiomyocytes were plated on cover slips, and, 90 min later, fixed in 3.5% paraformaldehyde for 10 min. The cell culture medium was removed, and the cells were washed three times with PBS, permeabilized in 0.1% Triton X-100 for 5 min at room temperature and washed again three times with PBS. This was followed by blocking with 10% horse serum and 0.01% azide in PBS for 2 h. Thereafter, the cells were incubated with a selective anti-Ca_v_1.2 antibody (#AGP-001, Alomone Labs; 1:500 in PBS) at 4°C overnight. The following day, cells were washed three times with PBS and incubated for 60 min with the corresponding secondary antibody (Alexa Fluor 594, #A11076, Invitrogen; 1:500 in PBS) at room temperature. After three subsequent PBS washing steps, the cells were mounted, dried and stored at 4°C. The slides were finally analyzed using a LSM 510 confocal microscope (Zeiss, Jena, Germany). For the immunostaining experiments, two wt and two *Dmd^mdx^* rats (9 months old) were used for cell isolation.

### Detection of L-type Ca^2+^ channel currents

Barium currents were recorded in the whole-cell mode of the patch-clamp technique from cardiomyocytes up to 6 h after preparation at an experimental temperature of 22±1.5°C, using an Axoclamp 200B patch-clamp amplifier (Axon Instruments, Union City, CA, USA). Pipettes were pulled from aluminosilicate glass (AF150–100-10, Science Products, Hofheim, Germany) with a P-97 horizontal puller (Sutter Instruments, Novato, CA, USA), and had resistances between 1 MΩ and 2 MΩ when filled with pipette solution (see below). Data acquisition was performed with pClamp 11.0 software (Axon Instruments) through a 16-bit A-D/D-A interface (Digidata 1440; Axon Instruments). Data were low-pass filtered with 2 kHz (3 dB) and digitized at 5 kHz. Leak currents and capacity transients were subtracted using a P/4 protocol. Data were analyzed with Clampfit 10.7 (Axon Instruments) and Prism 5.04 (GraphPad Software) software. For rapid solution exchange, a DAD-8-VC superfusion system (ALA Scientific Instruments, Westbury, NY, USA) was used. The bath solution contained 10 mmol/l BaCl_2_, 145 mmol/l TEA-Cl, 10 mmol/l HEPES, pH 7.4 adjusted with tetraethylammonium hydroxide solution. The pipette solution consisted of 145 mmol/l Cs-aspartate, 2 mmol/l MgCl_2_, 10 mmol/l HEPES, 0.1 mmol/l Cs-EGTA, 2 mmol/l Mg-ATP, pH 7.4 adjusted with CsOH. The currents were elicited from a holding potential of −80 mV by depolarizing voltage steps up to +50 mV. For the determination of current density-voltage relations, the current amplitudes at various voltages were measured and then divided by the cell capacitance to obtain current densities. The values were then plotted against the respective test pulse potentials. The kinetics of barium current inactivation was derived from single exponential fits of the current decay after channel activation at different membrane potentials, and expressed as τ-values.

### Statistical analyses

Data are expressed as means±s.d. The echocardiographic data were compared by unpaired two-tailed Student's *t*-test between groups at defined age (3, 7 and 9 months). Vascular relaxation in response to ACh or SNP was expressed as a percentage of contraction induced by PE. The statistical comparison between the relaxation and contraction responses was assessed using two-way analysis of variance (ANOVA) for repeated measures. Statistical comparisons between wt and *Dmd^mdx^* cardiac and EC gene expression values, and between wt and *Dmd^mdx^* cardiomyocytes, were made using an unpaired two-tailed Student's *t*-test. *P*<0.05 was considered significant.

## Supplementary Material

Supplementary information
