# Supplementary material for: Cardiovascular phenotype of the Dmdmdx rat – a suitable animal model for Duchenne muscular dystrophy
Source: Dis Model Mech. 2021 Feb 22;14(2):dmm047704. doi: 10.1242/dmm.047704 (PMC7927653; doi:10.1242/dmm.047704)
Supplement: Supplementary information [file dmm-14-047704-s1.pdf]

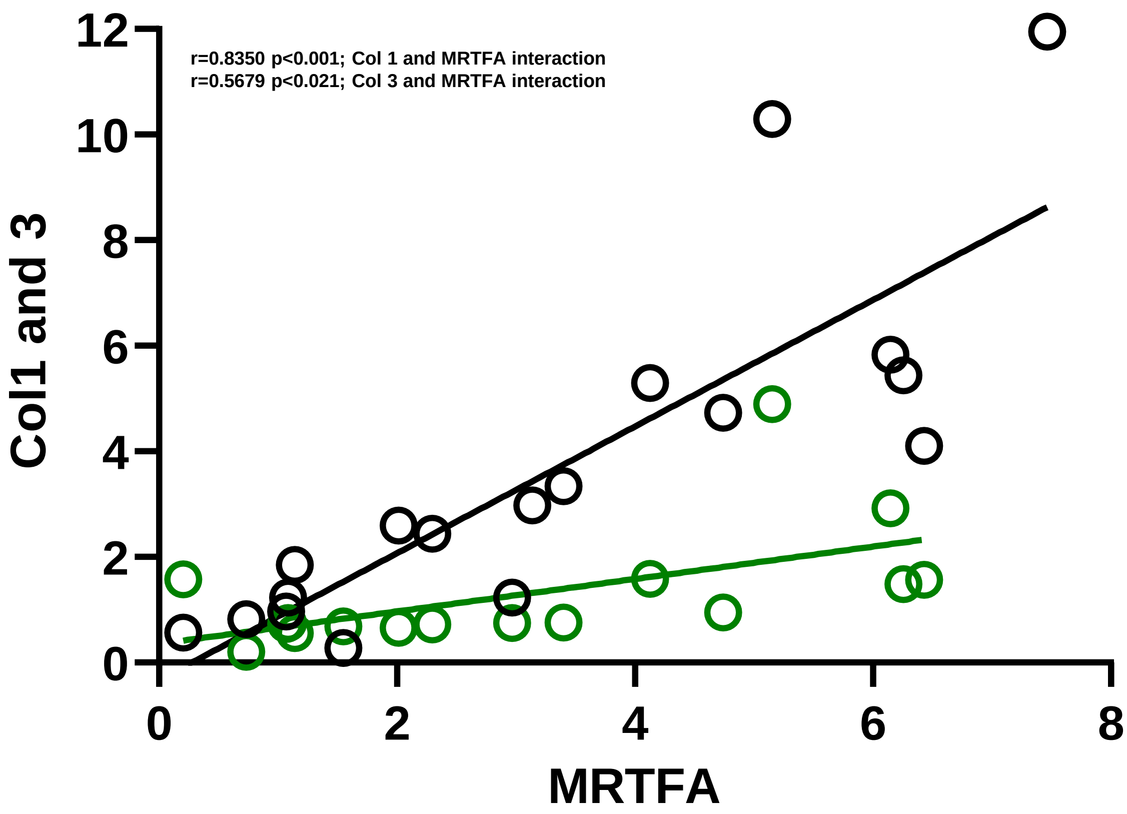

Figure S1.

Plots showing the correlation between Col 1 (black), Col 3 (green) and MRTFA mRNA expression, detected by RT-qPCR in cardiac tissue samples from wt and in *Dmd*^{mdx} rats at 9 months of age. Simple linear regression was used between the expression of Collagen I, III and MRTFA; n=10 wt and n=8 *Dmd*^{mdx}.

Disease Models & Mechanisms • Supplementary information

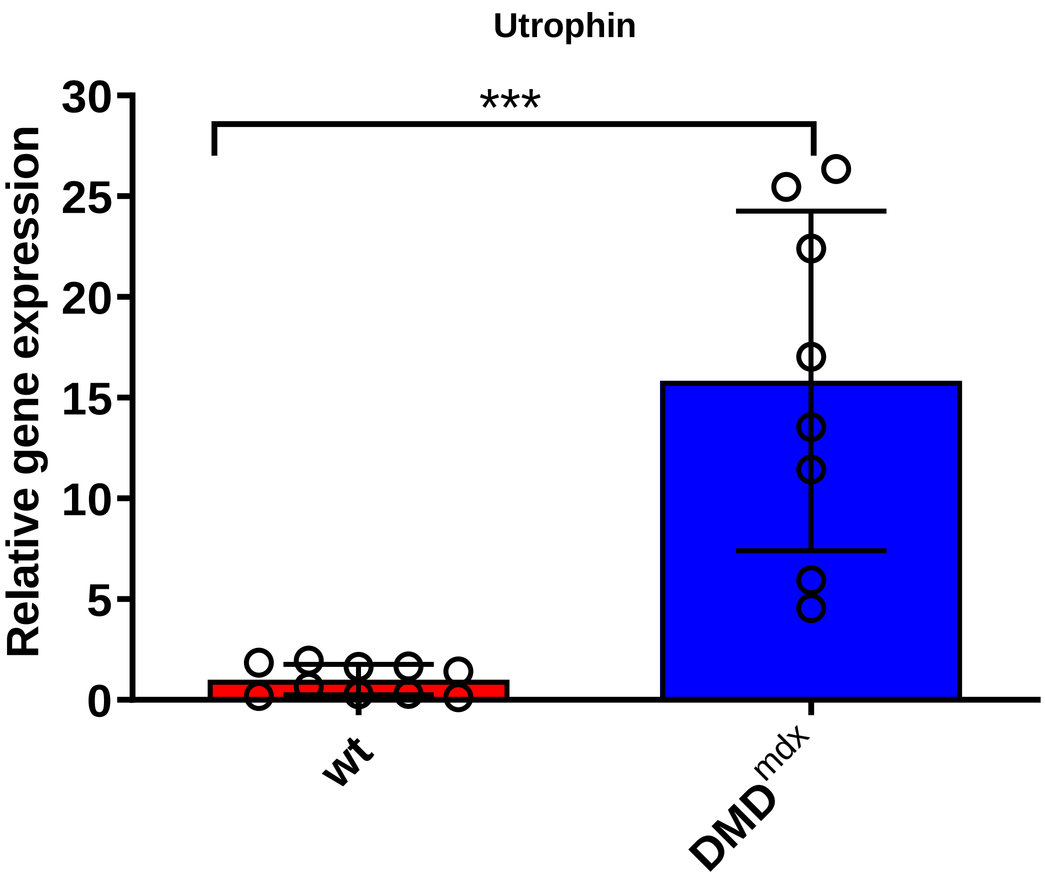

Figure S2.

mRNA expression of utropin in left ventricular tissue samples from wt and in *Dmd*^{mdx} rats at 9 months of age. Data are expressed as mean ±SD, n=10 wt and n=8 *Dmd*^{mdx}. **p<0.001 using unpaired Student's test.

Table S1. List of primer sequences

Gene	Forward 5'-3'	Primer lenght (bp)	Reverse 5'-3'	Primer lenght (bp)	Product (bp)
s18 (HKG)	CATTCGAACGTCTGCCCTAT	20	GTTTCTCAGGCTCCCTCTCC	20	194
SOD1	TTTTGCTCTCCCAGGTTCCG	20	CTGCTCGAAGTGAATGACGC	20	116
IL-1β	CAGGGAGGGAAACACACGTT	20	CCTTGTCGAGAATGGGCAGT	20	105
VCAM 1	AATGGGGAGGTGAGGGATGAAG	22	ACACATTAGGGACCGTGCAG	20	226
Col I	GGAGAGTACTGGATCGACCCTAAC	25	CTGACCTGTCTCCATGTTGCA	21	100
Col III	GAAAAAACCCTGCTCGGAATT	21	GGATCAACCCAGTATTCTCCACTCT	25	111
ACE1	TCCCAAGCGTGACACCATAC	20	CACACTGGTGTAGTGGACCC	20	111
AT_1R	TTCGTGGCTTGAGTCCTGTT	20	GGTGATCACTTTCTGGGAGGG	21	156
NOX4	TTTATTGGGCGTCCTCGGTG	20	TGGGTCCACAGCAGAAAACT	20	91
MRTFA	AGTCTTTCTATGCCCCTCCC	20	ACACCAACTCTCCACCCTTC	20	158
MRTFB	CCTTCCCAGTTCTTGTGTTC	20	TGTTCTTGTTCCAGTTTCCTC	21	173
PLN	GCCTCGACTATTGAAATGCCCC	22	ATGATGCAGATCAGCAGCAGAC	22	101
SERCA2	ACAAGCCAGTGAAATGCC	18	TCCAACTTTTTCATACACACCC	22	119
SLN	TTCAGGACGTGAAGACGAG	19	GTCAACAGAGCAGTGGAAG	19	238
UTR	GTGCTGCCCTTCAACACTTG	20	GGTATTACGCCAGTCCTCGG	20	116

s18: 18S ribosomal RNA, SOD1: superoxide dismutase 1, IL-1β: Interleukin-1 beta, VCAM: vascular cell adhesion molecule, Col I: collagen I, Col III: collagen III, ACE1: angiotensin converting enzyme 1, AT_1R: angiotensin receptor 1, NOX4: nicotinamide adenine dinucleotide phosphate oxidase 4, MRTFA: myocardin related transcription factor A, MRTFB: myocardin related transcription factor B, PLN: phospholamban, SERCA2: sarcoplasmic reticulum Ca ATPase, SLN: sarcolipin, UTR: utrophin

Disease Models & Mechanisms • Supplementary information
